# Exposing Empirical Links between COVID-19 Situation Report and Available Data: The Case of Nigeria

**DOI:** 10.3390/diseases8040038

**Published:** 2020-10-20

**Authors:** Yusuf F. Zakariya

**Affiliations:** Department of Mathematical Sciences, University of Agder, 4630 Kristiansand, Norway; yusuf.zakariya@uia.no

**Keywords:** COVID-19, Nigeria, low number of testing, correlational study, African most populous nation, pandemic, environmental factors

## Abstract

Ever since the index case of COVID-19 was announced in Nigeria, the number of confirmed cases has gradually increased to 46,140 (about 4.5% of total confirmed cases in Africa) as the time of writing this article. This seemingly low number of confirmed cases has provoked heated debates among researchers. This cross-sectional study explores the Nigerian COVID-19 report to expose some links between the number of confirmed cases, testing, and some environmental conditions. The findings reveal that there is no state in Nigeria which has up to 12 confirmed cases per 10,000 population. That means, the number of confirmed COVID-19 cases is less than 0.15% of the population of people across each state in Nigeria. On the flip side, it was revealed that the proportion of samples tested for COVID-19 is low compared to the population. The percentages ranging from less than 0.1% to a maximum of 0.7% of each state population in which 23 states out of the 37 states are within the less than 0.1% range. Furthermore, there is a substantial correlation (ρ (37)=0.903, p< 0.001) between the confirmed cases and testing. In contrast, no substantial correlation was found between the former with either average temperature or rainfall.

## 1. Introduction

There came a novel coronavirus (caused by severe acute respiratory syndrome coronavirus-2) at the tail end of the year 2019, when the world seemed to feel that the year was over and was looking forward to the new year 2020. Unknowingly to the world, the coronavirus disease (otherwise called COVID-19) has come to disrupt world activities by threatening our lives and livelihoods. The World Health Organization (WHO) declared COVID-19 a pandemic in the third week of March 2020 [1]. According to the COVID-19 Dashboard by the Center for Systems Science and Engineering (CSSE) at Johns Hopkins University, at the time of writing the present article, there were over 19.6 million confirmed cases of COVID-19 patients in over 200 countries and territories around the world. With these figures, one could be tempted to say if there was a more stringent word then a pandemic, then COVID-19 should be characterised as such. The virus has devastated many countries and exposed the weaknesses of several health systems in many countries. It has changed our lifestyles and thrown the world economy into disarray with significant job losses and bankruptcy in many big companies. Many lives have been lost (over 700,000 deaths around the world at the time writing the present article) and many relationships have been torn apart. This virus seems to be the only thing in recent times that has exposed the high level of human life insecurity on this planet, despite billions of United States dollars spent on arms and ammunitions yearly by several countries.

According to the latest count by the Africa Centres for Disease Control and Prevention (CDC), there are over one million confirmed cases of COVID-19 patients and 22,491 deaths in 55 African countries [2]. Surprisingly, Africa’s most populous nation, Nigeria [3], has only accounted for about 4.5% (i.e., 46,140) of the total confirmed COVID-19 cases in Africa, as reported by the Nigeria Centre for Disease Control (NCDC) on their official website https://covid19.ncdc.gov.ng/. On the one hand, these low confirmed cases in a country of over 200 million people call for commendations of whatever effort the government of Nigeria is using to curb the spread of the virus. For instance, shortly after the official pronouncement of the Nigerian COVID-19 index case in late February 2020, the Nigerian government set up a presidential task force (PTF) committee on COVID-19 that includes the minister of health, minister of aviation, minister of foreign affairs, director-general of the NCDC, minister of humanitarian affairs, disaster management and social development, minister of state for education, and the secretary to the government of the federation. The PTF committee is charged with the responsibility of coordinating, managing, and leading the government effort in combatting the virus around the country. Following the recommendations of the PTF committee, the Nigerian international borders were closed in late March, followed by a ban on inter-state travels, and the lockdowns of state economies. Both public and private offices and points of mass gathering were closed, including religious institutions and schools, from nurseries to tertiary institutions. These proactive measures might explain the low number of confirmed COVID-19 reported cases in the country. Nonetheless, some researchers, e.g., [3], have expressed some reservations about these measures and linked some of them (e.g., the lockdowns) to increased mental health problems in the country. Moreover, the lockdown was gradually eased in early May 2020, not because Nigeria experienced a decline in the number of confirmed COVID-19 cases but because the government wished to save the country’s economy from collapsing.

On the other hand, the low number of confirmed COVID-19 cases in Nigeria call for serious concern with the proper monitoring of the infection in terms of identifying, testing, tracing, isolating, and treating COVID-19 patients in the country. There are many questions that revolve around the identification, testing, tracing, isolating, and treating of COVID-19 patients. For instance, one can ask the question of how fast is the Nigerian government in identifying COVID-19 patients? How many COVID-19 tests have been conducted to date after six months of the infection in Nigeria? Is the Nigerian government engaging a blind-folded fight against the virus? Is Nigeria directly or indirectly leveraging on herd immunity [4] against the virus? How many contacts of COVID-19 infected persons have been traced by the Nigerian government? How quickly and responsibly are confirmed COVID-19 infected patients isolated and treated? What is the Nigerian government doing differently from the rest of the world in combatting the virus? Can the Nigerian temperate weather be linked to the reported confirmed COVID-19 cases? How many confirmed COVID-19 patients have been managed and treated successfully? How many people have been lost to the virus? The list of these questions can be elongated into several pages of this article. However, one must stop at a point.

Previous studies have attempted to address some of these questions using different approaches. For instance, Osseni [5], in an opinion letter to an editor of a journal, explored the level of preparation of African countries for the virus (lessons from a previous outbreak such as Ebola), their strategic responses (such as the creation of taskforces), and he exposed some hidden potential such as an opportunity to promote alternative medicines in treating the virus. Some other researchers, e.g., [6], have capitalised on lessons from previous disease outbreaks in Africa to offer recommendations for strengthening the African responses to COVID-19. Other researchers have explored the available knowledge of people, the attitudes of pharmaceutical practitioners, doctors and nurses toward their patients during the pandemic [7,8]. There have also been some predictions modelling results on the spread of the virus and treatment procedures (including non-pharmaceutical interventions such as social distancing and the use of facemasks) in Africa which include Nigeria as a case study [9,10]. However, none of these previous studies have conducted an in-depth analysis of the links between data generation processes and reported COVID-19 situations by the Nigerian government. Admittedly, most of the contextual data for Nigeria are difficult to assess, if available. In fact, a detailed breakdown of COVID-19 number of tests conducted by each state in Nigeria was reported, for the first time since the inception of COVID-19 infection, by the NCDC on 4 August 2020 [11].

As such, the present observational study explores the links between the available Nigerian data from multiple sources and a COVID-19 situation report in Nigeria. Attempts were made to expose these links in lieu of addressing some questions that surround the seemingly low reported number cases of COVID-19 infections despite the teaming and densely populated nature of the country. Or put differently, the present study attempts to address the following research question: what are the links between Nigerian data and the situation report on identifying, testing, and treating COVID-19 patients in Nigeria? By taking an objective look at the available data, it is expected that such links can be exposed. A holistic view was ensured in the present study such that data were consolidated from every part of the country to expose the purported links. It was presumed that the findings of the present study will provide potential cues to the Nigerian government on the right places to intensify their effort in combatting the virus. The PTF committee on COVID-19 will benefit from the findings of the present study in providing advice to the presidency on the next line of action in combatting the virus. More importantly, the findings of the present study will provide proper guidance to Nigerians in the diaspora or foreign visitors to Nigeria on the risk associated with visiting any part of the country. Finally, from the findings of the present study, it is envisaged that researchers and other public health stakeholders will have a clear understanding of the Nigerian COVID-19 situation report.

## 2. Materials and Methods

### 2.1. Research Design

In the present study, a cross-sectional research design was adopted [12]. This design involved an observational research procedure in which data were generated or extracted from multiple sources and integrated to explore the patterns of relationships between the data. The researcher ensure negligible biases during the integration of the data and avoided the manipulation of the research variables. It was believed that for an objective view of the data to be maintained, the cross-sectional research design was more appropriate for the present study, instead of other competing research designs such experimental designs, longitudinal designs and comparative designs [12].

### 2.2. Study Context

Nigeria is a multi-ethnicity multi-language West African country that spans an area of 923,768 square kilometres, with an estimated population of over 200 million people [13]. Nigeria is made up of six geopolitical zones (South West, North East, South East, North West, North Central, and South South) which encompass 36 states and the Federal Capital Territory (FCT). The South West comprises six states which are Lagos, Oyo, Ondo, Ogun, Osun, and Ekiti. The North East comprises six states which are Taraba, Bauchi, Yobe, Gombe, Borno, and Adamawa. The South East comprises five states which are Anambra, Abia, Enugu, Imo, and Ebonyi. The North West comprises seven states which are Zamfara, Kano, Katsina, Kaduna, Sokoto, Kebbi, and Jigawa. The North Central comprises six states which are Kwara, FCT, Kogi, Niger, Nasarawa, Plateau, and Benue. The South South comprises six states which are Akwa Ibom, Edo, Rivers, Cross River, Delta, and Bayelsa [13].

### 2.3. Data Sources

The data used for the present study were generated from different sources. For instance, the Nigerian data for the total number of confirmed COVID-19 cases per state, and the total number of samples tested for COVID-19 in each state including the FCT, were sourced from a situation report published by the NCDC on the 4 August 2020 [11]. The 4 August situation report by the NCDC was used in the present study because it was the latest report by the NCDC that contained a breakdown of the total samples tested for COVID-19 for each state including the FCT. The technique of national collation of the number of confirmed COVID-19 cases followed from the COVID-19 task force in each state. Each state in Nigeria, including the FCT, has a COVID-19 task force committee that is responsible for the state response on sample collection, testing, contact tracing, and the treatment of COVID-19 patients in the state. These state COVID-19 task forces report the statistics of the COVID-19 situation in their state to the NCDC for a national response on the virus. Moreover, the data on the average amount of rainfall and temperature were generated using online sources with some levels of reliability as perceived by the researcher.

### 2.4. Projected Population

Another important variable in the present study was the projected population of Nigeria. Unfortunately, the National Population Commission of Nigeria does not make public any estimated population of the country for the year 2020. The National Population Commission conducted the last population census in the country in the year 2006 [14]. Ever since then, researchers and other government agencies have relied on a projected population of Nigeria using one model or the other. In the present study, I adopted the exponential model of the National Bureau of Statistics (given by Equation (1)) to estimate the 2020 population for each state in Nigeria, including the FCT:

(1) $$Projected\;population\;=ae^{bn}$$

In Equation (1), a is the 2006 population for each state, including the FCT, as reported by the National Population Commission in the year 2006 [14]. The constant b is a fixed annual population growth rate (an index that is provided by the National Bureau of Statistics for each state in Nigeria, including the FCT). Finally, n is the number of years from the initial population year of 2006, and e is the exponential constant with an approximate value of 2.781 (3 significant figures). For instance, Abia state in the South East of Nigeria has a population of 2,845,380 and an annual population growth rate of 0.027 [14]. Hence, with the help of Equation (1), a projected population of Abia state for the year 2020 (14 years later) is estimated as follows:

(2) $$Projected\;population_{Abia}=2,845,380\;\times \;e^{0.027\;\times \;14}=4,152,442$$

Similar computations were done to obtain projected populations for each state in Nigeria, including the FCT as presented in Table 1. It is important to remark that the computations are carried out with an assumption that the population growth rates for each state and the FCT remain constant since the year 2006. It was acknowledged that this assumption was fallible, and as such, the projected populations were most likely going to be biased downward. However, these underestimated populations for each state in Nigeria, including the FCT, seem to be the best estimate available across every state of the federation, including the FCT.

### 2.5. Procedures for Data Analysis

The generated data were analysed using basic descriptive statistics that involved line graphs and Pearson correlations. On the one hand, the line graphs were used to present graphical displays of some distributions across the 36 states and the FCT. The graphical displays afford an opportunity to present a clear view of the trends of events across the country as per the situation of COVID-19. On the other hand, Pearson correlations were used to investigate the relationship between the variables of the study. Pearson correlations were considered the most appropriate correlation coefficient, given that all the variables of the present study were at a ratio level of measurement.

## 3. Results and Discussions

### 3.1. Confirmed COVID-19 Cases per State Population

The first set of results in the present study concerns the distribution of the confirmed COVID-19 cases across the 36 states and the FCT in Nigeria. As of 4 August 2020, a total of 44,433 confirmed COVID-19 cases were reported by the NCDC across the 36 states and the FCT [11]. A confirmed case is “a person with laboratory confirmation of SARS-CoV-2 infection with or without signs and symptoms” [15]. By laboratory confirmation, the NCDC intends a polymerase chain reaction (PCR) test that returns positive for the nasal swab and oropharyngeal swab samples tested [15]. There are some guidelines outlined by the NCDC through which a person is qualified to be tested for the virus. People with symptoms such as a cough, fever, difficulty in breathing and that are arriving passengers from a country with confirmed cases of COVID-19, people in close contact with a confirmed case, and people in close contact with health facility where a COVID-19 case has been confirmed are qualified to be tested for the virus. These people are classified as suspect cases [15]. If their tests results return positive, they are either admitted to government COVID-19 facilities in each state or treated at home if such facilities are not available. As for confirmed COVID-19 cases without symptoms, they are expected to self-isolate, in most instances, for 14 days, pending a negative COVID-19 test of such patients.

**Figure 1:**
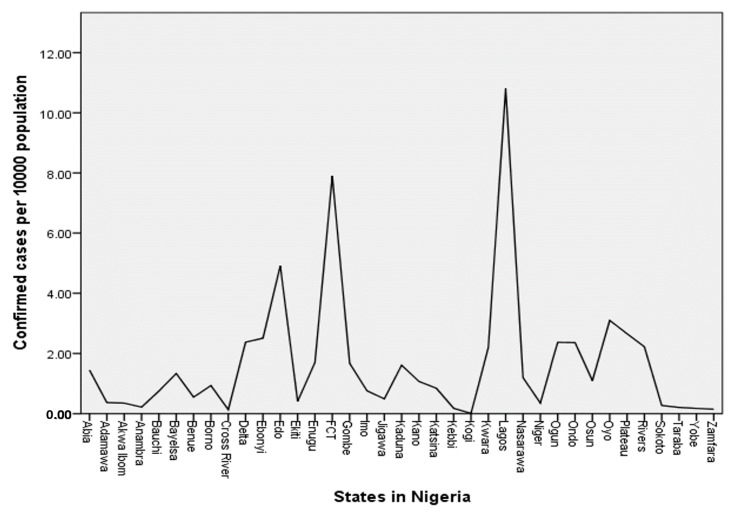
COVID-19 confirmed rates per 10,000 population of each state and the FCT in Nigeria.

For a clear understanding of the number of confirmed cases, I explored the total number of confirmed COVID-19 cases per 10,000 projected population of each state against the respective states and the FCT. Information from Table 1 and data from the NCDC 4 August 2020 report, were used for this analysis—the results of which are presented in Figure 1. Given that the projected population of Nigeria for the year 2020 is 220,384,426, one can compute the COVID-19 infectious rate per 10,000 population (henceforth, infectious rate) of the country to be approximately 2. That is, two people out of a total of 10,000 people in Nigeria have tested positive for COVID-19.

The presented results in Figure 1 show the distribution of confirmed COVID-19 cases per 10,000 population of states and the FCT in Nigeria. Lagos state, with approximately 11 confirmed cases out of 10,000 people in the state, has the highest infectious rate in Nigeria, followed by the FCT with an approximate infectious rate of 8. Both the Lagos state and the FCT obviously have higher infectious rates than the national infectious rate of 2. Other states with higher infectious rates than the national infectious rate are Edo (4.9), Oyo (3.1), Plateau (2.7), Ebonyi (2.5), Delta (2.4), Ogun (2.4), Ondo (2.4), Rivers (2.2), and Kwara (2.2). What can be deduced from these numbers across the 36 states and the FCT in Nigeria? Eight people in Lagos state, for instance, have tested positive for the virus out of 10,000 people in the state. Can COVID-19 be described as an epidemic disease in the state with this figure? One can also take Kwara state in the North Central, for instance, with approximately two people out of 10,000 people testing positive for the virus. At the tail end of the infectious rate distribution in Nigeria (Figure 1) are Sokoto, Anambra, Taraba, Kebbi, Yobe, Zamfara, Cross Rivers, and Kogi with infectious rates of less than 0.25. That is, to identify one person that is a confirmed case of COVID-19, one needs to accumulate at least 40,000 people in each of these states. How accurately do these data reflect the situation of the COVID-19 pandemic in the country?

### 3.2. COVID-19 Tests per State Population

**Figure 2:**
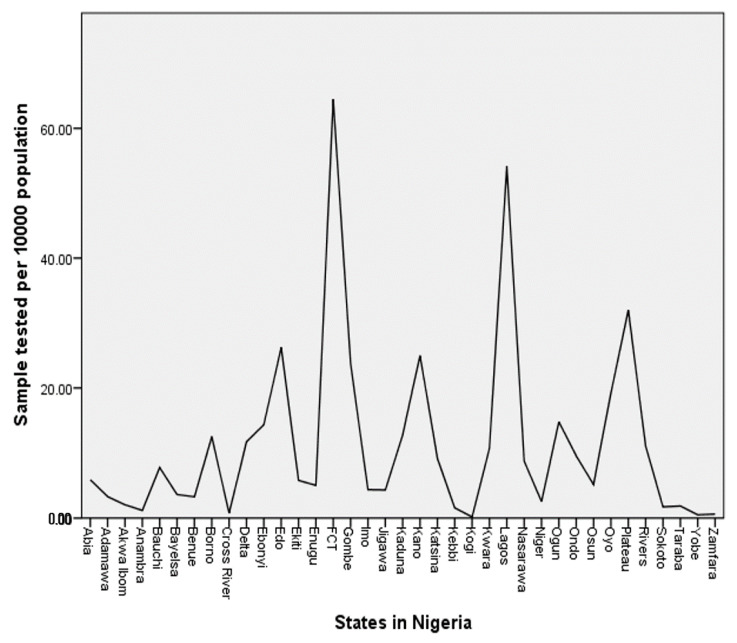
COVID-19 test rates per 10,000 population of each state and the FCT in Nigeria.

I turn to the number of the PCR tests conducted per 10,000 population (henceforth, test rate) of each state and the FCT in Nigeria. According to NCDC [11], the total number of samples tested using PCR as of 4 August 2020, across Nigeria, was 304,221. One can compute the test rate of the country by dividing this total number of samples tested with the projected population of Nigeria (Table 1) and then multiply it by 10,000. This computation shows the test rate to be 13.8 for the country. That is, out of 10,000 people in the Nigerian population, the government tested approximately 14 people for COVID-19, which is less than 0.2% of the population. For a better understanding of the test rate across each state and the FCT in Nigeria, I present the distribution of numbers in Figure 2.

The presented results in Figure 2 show the distribution of the COVID-19 test rate of the 36 states and the FCT in Nigeria. The FCT with a test rate of 64.5 conducted the highest number of COVID-19 tests per 10,000 population in Nigeria. The second highest rate was in Lagos state with a test rate of 51.2. Both the FCT and Lagos state obviously conducted more COVID-19 tests than the national test rate of 13.8. Other states with higher test rates than the national rate were Plateau (40), Edo (26.3), Kano (25), Gombe (23.7), Oyo (19.2), Ogun (14.8), and Ebonyi (14.3). Unfortunately, none of these states, including Lagos state and the FCT, tested up to 0.7% of their population for COVID-19. The results in Figure 2 also revealed that the following states have less than 0.2 testing rates: Taraba, Sokoto, Kebbi, Anambra, Cross Rivers, Zamfara, Yobe, and Kogi. That is, these states have tested less than 0.002% of the population of people in their states. To be more specific, only three states have tested between (0.3 and 0.7)% of their population, 11 states have tested between (0.1 and 0.3)% of their population, while the remaining 23 states have tested less than 0.1% of their population. I believe that these figures call for serious concern among stakeholders in Nigeria. A pandemic like COVID-19 cannot obviously be curbed without adequate testing [16]. To substantiate this fact, the researcher explores the correlation the between test rate and infectious rates across the 36 states and the FCT in Nigeria. The results are presented in Table 2.

### 3.3. Correlation between Test Rate and Confirmed COVID-19 Cases Per State Population

Table 2 shows the Pearson correlation coefficient (ρ) between the infectious rates and test rates across the 36 states and the FCT in Nigeria. The statistics show that the correlation coefficient is significant, ρ (37)=0.903, p< 0.001. This result means that there is a substantial non-trivial relationship between the number of PCR tests conducted per 10,000 population of each state, including the FCT and the number of confirmed COVID-19 cases per state population. In specific terms, 81.5% (i.e., $\rho^{2}\times 100)\;$of variances in the number of confirmed COVID-19 cases per state population is shared with the number of PCR tests conducted per 10,000 population of each state including the FCT. This finding shows that an important factor that contributes substantially to the seemingly low number of reported confirmed COVID-19 cases in Nigeria is the number of PCR tests conducted in the country. One may argue that the variation between the number of confirmed COVID-19 cases per state population is due to some varied weather conditions, such as the temperature and amount of rainfall across the state. The argument seems plausible considering the fact that some studies have reported non-trivial correlations between environmental conditions and the spread of COVID-19 [17,18]. Moreover, there have been some speculations in the country on the relationship between the number of confirmed COVID-19 cases and varied weather conditions such as the temperature and amount of rainfall across the 36 states of the federation and the FCT. To clear the air on this matter, the researcher explores this possibility of correlating the infectious rates with the amount of rainfall and temperature across the 36 states and the FCT in Nigeria. The results of these analyses are presented in Table 3.

### 3.4. Correlation between Environmental Conditions and Confirmed COVID-19 Cases per State Population

The presented results in Table 3 show the Pearson correlation coefficients between COVID-19 infection rates, the amount of rainfall in millimetres, and temperature in °C. The amount of rainfall is the annual average rainfall per each state and the FCT in Nigeria whose data are sourced from some weather forecast websites. Additionally, the temperature is the annual average temperature per each state and the FCT in Nigeria whose data are sourced from some weather forecast websites. Table 3 shows that the one-tailed correlation coefficient between the amount of rainfall and the infectious rates is not significant, ρ (37)=0.199, p=0.120. In a similar manner, the table shows that the one-tailed correlation coefficient between the temperature and the infectious rates is negative and not significant, ρ (37)=−0.104, p=0.271. These results may be interpreted to mean that neither the annual average rainfall nor the annual average temperature per each state and the FCT in Nigeria has a substantial association with the reported number of confirmed COVID-19 cases across the states of the federation. As such, the results strengthen the fact that a major contributing factor to the variances in the reported confirmed COVID-19 cases across the 36 states and FCT in Nigeria remains the number of COVID-19 tests.

## 4. Conclusions

The ugly situation of COVID-19 around the world is reverberating in the minds of millions of people through threats of lives and livelihoods. The African continent is not spared with over one million confirmed cases at the time of writing the present article. Surprisingly, the reported confirmed cases of COVID-19 infections in the most populous African country appear to be low, with 46,140 (about 4.5% of total confirmed cases in Africa) cases at the of writing. The present study focuses on Nigerian data in an attempt to uncover the links between the reported cases of COVID-19 infections, the number of COVID-19 tests conducted, and some environmental conditions such as the amount of annual rainfall and annual average temperature across the 36 states and the FCT in the country. The findings of the present study reveal some interesting facts that are worthy of emphasising.

At first, the distribution of confirmed COVID-19 cases per 10,000 population seems unalarming, since no single state in Nigeria, including the FCT, has up to 12 confirmed cases per 10,000 population. This means that the number of confirmed COVID-19 cases is less than 0.15% of the population of people across each state and the FCT in Nigeria. Additionally, the distribution (Figure 1) might serve as a guide for visitors to Nigeria to determine the high COVID-19 risk areas across the 36 states and the FCT. However, an important question surrounds the extent to which the distribution (Figure 1) reflects the situation of COVID-19 infection in Nigeria. To gain an insight into how reflective the reported COVID-19 cases of the Nigerian situation are, the researcher explores the distribution of the number of COVID-19 tests across the states of the federation. The findings reveal that Nigeria has only tested 14 out of 10,000 people in the country for COVID-19. That is, less than 0.2% of the Nigerian population has been tested for the virus over a span of six months of the pandemic. For each state, and the FCT, the percentages range from less than 0.1% to a maximum of 0.7% of the state population in which 23 states out of the 36 states and the FCT are within less than 0.1% range. Given that several studies, e.g., [16,19,20,21], have underscored the importance of mass testing in fighting the virus, one may conclude that the test rates across Nigeria are low compared to the population. Therefore, it is recommended that the government of Nigeria and other public health stakeholders should improve the testing capacity and the number of samples tested for the virus across the country.

Another crucial finding of the present study is the high correlation coefficient that was found between the number of confirmed COVID-19 cases and the number of samples tested for the virus. The finding shows that about 82% variances in the former are shared with the latter. This finding corroborates the findings of previous studies e.g., [5,6] that have identified inadequate testing capacity as one of the difficulties faced by African countries in combating the virus. As rightly put in the following quote, “you cannot fight a fire blindfolded. And we cannot stop this pandemic if we don’t know who is infected.” (World Health Organization Director-General, 16 March 2020). The Nigerian government may consider the use of rapid test kits [19] to supplement the PCR testing that is currently in use across the country. Moreover, contrary to the report by Tosepu, Gunawan [17], the correlation coefficient between the number of confirmed cases and the average temperature across the 36 states and the FCT in Nigeria is not substantial. Similarly, no substantial correlation coefficient was also found between the number of confirmed cases and the average amount of rainfall across the 36 states and the FCT in Nigeria. Hence, both the average annual temperature and amount of rainfall have trivial associations with variations in the number of confirmed COVID-19 cases across the 36 states and the FCT in Nigeria.

Finally, it is important to remark that there are some other factors such as a lack of testing laboratories, attitudes of people to testing, and the stigmatisation of COVID-19 patients that may contribute to low testing in the country. The Nigerian government needs to intensify efforts in improving these factors. Furthermore, the present study is purely observational in that it only presented provisional evidence on links between the available data. The researcher does not make or evaluate any causal claims in this study. Moreover, as another limitation of the present study, some data such as the year 2020 population projections may bias the results. Caution must be taken in interpreting these results. As such, future studies are recommended to complement the findings reported herewith. By implications, the findings of the present study expose some links and gaps that surround situation report on mostly identifying, and testing COVID-19 patients in Nigeria. Nigerians in Nigeria, government officials, visitors to Nigeria, international observers, researchers, and global public health stakeholders will benefit from the findings of the present study.

## Figures and Tables

**Table 1:** The projected population for each state in Nigeria including the Federal Capital Territory (FCT) for the year 2020.

State	Population (2006)	Annual Growth Rate	Projected Population (2020)
Abia	2,845,380	0.027	4,152,442
Adamawa	3,178,950	0.029	4,770,976
Akwa Ibom	3,902,051	0.034	6,280,831
Anambra	4,177,828	0.028	6,182,925
Bauchi	4,653,066	0.034	7,489,682
Bayelsa	1,704,515	0.029	2,558,140
Benue	4,253,641	0.03	6,473,878
Borno	4,171,104	0.034	6,713,905
Cross River	2,892,988	0.029	4,341,804
Delta	4,112,445	0.032	6,436,711
Ebonyi	2,176,947	0.028	3,221,746
Edo	3,233,366	0.027	4,718,655
Ekiti	2,398,957	0.031	3,702,595
Enugu	3,267,837	0.03	4,973,522
Gombe	2,365,040	0.032	3,701,710
Imo	3,927,563	0.032	6,147,338
Jigawa	4,361,002	0.029	6,545,003
Kaduna	6,113,503	0.03	9,304,517
Kano	9,401,288	0.033	14,922,150
Katsina	5,801,584	0.03	8,829,788
Kebbi	3,256,541	0.031	5,026,207
Kogi	3,314,043	0.03	5,043,846
Kwara	2,365,353	0.03	3,599,976
Lagos	9,113,605	0.032	14,264,420
Nasarawa	1,869,377	0.03	2,845,120
Niger	3,954,772	0.034	6,365,692
Ogun	3,751,140	0.033	5,953,979
Ondo	3,460,877	0.03	5,267,322
Osun	3,416,959	0.032	5,348,151
Oyo	5,580,894	0.034	8,983,135
Plateau	3,206,531	0.027	4,679,493
Rivers	5,198,716	0.034	8,367,973
Sokoto	3,702,676	0.03	5,635,331
Taraba	2,294,800	0.029	3,444,042
Yobe	2,321,339	0.035	3,789,159
Zamfara	3,278,873	0.032	5,132,022
FCT	1,406,239	0.093	5,170,238
Nigeria	140,431,790	0.032	220,384,426

**Table 2:** Pearson correlation between COVID-19 infectious rates and test rates across 36 states and the FCT in Nigeria.

	Mean	Standard Deviation	Number of States Including the FCT	Pearson Correlation Coefficient	*p*-Value
Infectious rates	1.669	2.173	37	0.903 *	<0.001
Test rates	11.407	14.139	37		

* Significant at *p* < 0.01 (two-tailed).

**Table 3:** Pearson correlations between COVID-19 infectious rates and weather conditions across 36 states and the FCT in Nigeria.

	Mean	Standard Deviation	Number of States Including the FCT	Pearson Correlation Coefficient	*p*-Value(One-Tail)
Infectious rates	1.669	2.173	37		
Amount of rainfall (mm)	1443.000	657.740	37	0.199	0.120
Temperature (°C)	26.368	1.0047	37	−0.104	0.271
